# Bioimpedance Spectroscopy for Assessment of Volume Status in Patients before and after General Anaesthesia

**DOI:** 10.1371/journal.pone.0111139

**Published:** 2014-10-31

**Authors:** Matthäus Ernstbrunner, Lisa Kostner, Oliver Kimberger, Peter Wabel, Marcus Säemann, Klaus Markstaller, Edith Fleischmann, Barbara Kabon, Manfred Hecking

**Affiliations:** 1 Medical University of Vienna, Department of Anaesthesiology and Critical Care Medicine, Vienna, Austria; 2 Fresenius Medical Care, Bad Homburg, Germany; 3 Medical University of Vienna, Department of Internal Medicine III, Clinical Division of Nephrology & Dialysis, Vienna, Austria; Medical University of Graz, Austria

## Abstract

**Background:**

Technically assisted assessment of volume status before surgery may be useful to direct intraoperative fluid administration. We therefore tested a recently developed whole-body bioimpedance spectroscopy device to determine pre- to postoperative fluid distribution.

**Methods:**

Using a three-compartment physiologic tissue model, the body composition monitor (BCM, Fresenius Medical Care, Germany) measures total body fluid volume, extracellular volume, intracellular volume and fluid overload as surplus or deficit of ‘normal’ extracellular volume. BCM-measurements were performed before and after standardized general anaesthesia for gynaecological procedures (laparotomies, laparoscopies and vaginal surgeries). BCM results were blinded to the attending anaesthesiologist and data analysed using the 2-sided, paired Student’s t-test and multiple linear regression.

**Results:**

In 71 females aged 45±15 years with body weight 67±13 kg and duration of anaesthesia 154±68 min, pre- to postoperative fluid overload increased from −0.7±1.1 L to 0.1±1.0 L, corresponding to −5.1±7.5% and 0.8±6.7% of normal extracellular volume, respectively (both p<0.001), after patients had received 1.9±0.9 L intravenous crystalloid fluid. Perioperative urinary excretion was 0.4±0.3 L. The increase in extracellular volume was paralleled by an increase in total body fluid volume, while intracellular volume increased only slightly and without reaching statistical significance (p = 0.15). Net perioperative fluid balance (administered fluid volume minus urinary excretion) was significantly associated with change in extracellular volume (r^2^ = 0.65), but was not associated with change in intracellular volume (r^2^ = 0.01).

**Conclusions:**

Routine intraoperative fluid administration results in a significant, and clinically meaningful increase in the extracellular compartment. BCM-measurements yielded plausible results and may become useful to guide intraoperative fluid therapy in future studies.

## Introduction

All patients undergoing anaesthesia and surgery routinely receive intravenous (iv) fluid therapy intraoperatively for correction of anaesthesia- and surgery-related hemodynamic disturbances. Timing, amount and the specific type of its administration, however, remain controversial [1]. Based on the assumption of preoperative dehydration and intraoperative ‘third space’ fluid loss, administering a considerable amount of iv fluid is still a common clinical practice, although positive perioperative fluid balance with postoperative fluid-based weight gain is associated with major complications [2]–[6]. Cardiac-, pulmonary-, renal- and gastrointestinal function, respectively tissue oxygenation, wound healing and coagulation are all influenced by perioperative fluid therapy [7].

Surgery represents a traumatic insult and initiates an acute stress response leading to secretion of proinflammatory mediators and stress hormones, which control the sympathoadrenal system and the hypothalamic-pituitary-adrenal axis [8], [9]. Increased levels of adrenocorticotrophic hormone, antidiuretic hormone, cortisol, aldosterone and catecholamines result in increased catabolism, reduced urinary secretion, disturbed microcirculation and increased vascular permeability leading to salt and fluid retention [10]. Perioperative fluid therapy represents an approach to counteract the incidence and severity of systemic inflammation following surgery [9].

Total body fluid volume (TBV) comprises approximately 50% of the female body mass, i.e. 30 liters in a 60 kg woman. Of this amount, about two-thirds (20 liters) are intracellular volume (ICV) and one-third (10 liters) extracellular volume (ECV) in a healthy, young subject [11]–[13]. The body composition monitor (BCM, Fresenius Medical Care, Germany) is a novel, whole-body bioimpedance spectroscopy device that measures TBV, ICV and ECV by using a three-compartment physiologic tissue model based on the expected normal volume status with healthy renal function in a normohydrated subject [14]. The BCM was originally developed to calculate fluid overload in patients undergoing renal replacement therapy [15]. Extensive validation of the fluid volumes and body composition obtained with BCM has been performed against reference methods (bromide-, deuterium dilution and total body potassium method) involving healthy volunteers and hemodialysis patients [14], [16], [17].

To the best of our knowledge, the BCM device has not previously been studied in patients undergoing general anaesthesia, although objective assessment of fluid status before surgery may be useful to optimize intraoperative fluid administration and to prevent postoperative fluid retention. The aim of the present study was to determine the pre- to postoperative fluid distribution using bioimpedance spectroscopy technology, and, by relating changes in volume status to the net administration of iv fluid, to evaluate if BCM-derived measurements yield plausible results.

## Materials and Methods

### Patients

The use of routine BCM-measurements was instituted at the Department of Anaesthesiology of the Medical University of Vienna, as of May 2012, based on the availability of a BCM device provided by the Department of Nephrology. From 12 November 2012 until 31 January 2013, BCM-measurements were consecutively performed directly before general anaesthesia in unselected, conscious females, presenting for various elective gynaecological procedures. Patients with a pacemaker, with a limb amputation or pregnant women were not measured. Patients who had significant blood loss and therefore had received erythrocyte concentrates or colloid solution, and/or all those patients who had not received a urinary catheter were measured, but excluded from the present analysis. All patients fasted overnight for more than 6 hours. All patients received isotonic crystalloid solution (Elo-mel, Fresenius Kabi Austria) continuously, throughout the duration of anaesthesia. The amount and timing of intraoperative fluid administered was at the discretion of the attending anaesthesiologist, who was not informed about the BCM result, thereby reflecting clinical practice, as influenced by cardiovascular signs and fluid losses, without following a fixed fluid regime.

For induction of general anaesthesia propofol, fentanyl and rocuronium were administered intravenously. To secure the airway, an endotracheal tube was inserted as appropriate. Anaesthesia was maintained with sevoflurane in an air/oxygen mixture or with a continuous propofol infusion guided by electroencephalogram (Narcotrend index, MT MonitorTechnik, Bad Bramstedt, Germany). At the discretion of the attending anaesthesiologist, and depending on the anamnesis of the patient, a serotonin antagonist (usually ondansetron), a glucocorticoid (usually dexamethasone) in low dose (4 mg) and/or a total intravenous anaesthesia (TIVA) were administered intraoperatively to prevent postoperative nausea and vomiting (PONV). Urinary catheterization was performed after induction. Body temperature was maintained with temperature controlling devices like Bair Hugger (3 M) and Hotline 2 Fluid Warmer (Smiths Medical). The second BCM-measurement was performed as soon as possible after the end of anaesthesia in the postanaesthesia care unit.

To evaluate the obtained data in the form of the present open, non-interventional, observational cohort study, we obtained approval from the Ethics Committee of the Medical University of Vienna (EK Nr.: 2059/2012) and anonymised all participant information. The study adhered to the Declaration of Helsinki.

### BCM-measurements

BCM-measurements were performed as proposed by the manufacturer. Briefly, four non-recyclable electrode strips were taped to wrist and ankle, respectively hand and foot, on one side of the patient body in supine position. Electrodes were connected to the BCM device using the cable provided by the manufacturer. Body weight and height, patient age and patient gender were entered into the BCM device.

About BCM technology: The measurement itself usually requires <2 minutes; the BCM device measures resistance and reactance at 50 discrete frequencies covering the frequency spectrum from 5 to 1000 kHz. Extracellular and intracellular resistance values are obtained on the basis of a Cole model [18]. Using these resistances values, ECV, ICV and TBV are calculated from a fluid model described by Moissl [17]. Excess extracellular fluid is distinguished from the physiological hydration of lean and adipos tissue, based on a physiological tissue model described by Chamney [14]. This algorithm is built into the BCM device and calculates the normal hydration status for a given weight, i.e. the expected normal values for ECV, which would result with healthy renal function and in a state of normohydration. Specifically, the tissue hydration status is calculated from the difference between the normal ECV expected, and with BCM measured ECV. Excess extracellular fluid (fluid overload: FO) is then indicated as ‘overhydration’ in liters and in percent above “normal” ECV on the BCM device. In dehydrated patients, the value for ‘overhydration’ indicated on the BCM device is negative.

Additional information provided by a typical BCM-measurement comprise basic measures of the body composition, such as lean tissue mass, lean tissue index (lean tissue mass/height^2^), fat mass and fat tissue index (fat tissue mass/height^2^).

Note that the terms fluid overload, volume overload and overhydration are often used interchangeably. Since ‘hydration’ refers strictly to water, while ‘volume expansion’ refers to the accumulation of isotonic fluid (salt and water), we primarily use the term fluid overload and fluid status. Similarly, the term extracellular water used in previous publications [19]–[22] has here been replaced by extracellular fluid volume (ECV).

### Other measurements and patient characteristics

The patient characteristics recorded for this study included age, height, body weight, body mass index, classification per the American Society of Anaesthesiologists’ (ASA) physical status classification system, and New York Heart Association (NYHA) functional classification. All biochemical and hematological analyses (labs) were performed by the Clinical Institute of Laboratory Medicine of the Medical University of Vienna (for methods, see www.kimcl.at). Preoperative measurements of hemoglobin, serum creatinine and gamma glutamyl transferase were available in all patients. Increased vascular permeability might have influenced the BCM results, and is associated with hypoalbuminaemia and inflammation (manifested by high C-reactive protein [CRP] levels). To assess vascular permeability we therefore calculated the capillary leak index as CRP [in milligrams per deciliter] over albumin [in grams per liter], multiplied by 100 [23].

Pre- and postoperative heart rate, blood pressure measurements and fluid balance data were documented in all patients. Measurements of body temperature with single-use oesophageal temperature probes from Dräger were available in 51 patients. Urinary output was recorded in all patients. Net perioperative fluid balance was calculated by subtracting urine output from infused fluid.

### Data analysis: Statistics and sample size considerations

Descriptive statistics (mean ± standard deviation and relative frequency) were used to portray patient characteristics, laboratory values, iv fluid administration and urinary output, as well as all BCM-derived measurements. Changes in BCM-derived measurements from pre- to postoperatively (delta values) were obtained by subtracting the preoperative value from the postoperative value.

The primary parameter of interest was fluid overload (FO in liters and in % ECV). The change in pre- to postoperative FO was evaluated using the paired, two-sided Student’s t-test, as proposed in the pre-specified statistical analysis plan of the study protocol. Similar exploratory analyses were conducted for all other parameters obtained before and after anaesthesia. When the distribution of the analyzed variables was tested by Kolmogorov-Smirnov test, fluid therapy was found to be non-normally distributed.

Sample size considerations were based on association analyses using the Pearson correlation test. To detect a moderate, and thereby clinically meaningful correlation (r = 0.4) between net perioperative fluid balance and change in FO, a sample of 62 analysable subjects was calculated to provide 90% power to discover that the correlation would be significantly different from there being no correlation (i.e. that the correlation would be near zero) at the 0.05 level.

We used multiple regression analysis to examine the effect of different predictor variables (net perioperative fluid balance, serum albumin, age, body mass index, serum creatinine, preoperative iv fluid therapy, preoperative blood pressure, pre- and postoperative capillary leak index) on the outcome variable (change in ECV).

For calculations we used MS Excel 2011 and IBM SPSS Statistics 22.0.

## Results

### Characteristics of the study population

The study population comprised 71 females, aged 45±15 years, with body weight 67±13 kg (Table 1) after excluding 26 patients without a urinary catheter, 2 patients who had received erythrocyte concentrates intraoperatively without obtaining colloids and 7 patients who had received colloid solution in addition to crystalloid solution. Of these 71 patients, 2 patients had evidence of severe systemic disease, per the ASA physical classification system, and 14 patients were classified as NYHA stage 2 patients. No patient had serum creatinine >1.2 mg/dL, indicating that renal function was not impaired. The gynaecological procedures were 23 laparotomies (32%), 43 laparoscopies (61%) and 5 vaginal operations (7%).

**Table 1:** Demographic characteristics of the study population.

	All
N Patients	71
Females	100%
Age [years]	45±15 (22; 71)
Height [cm]	163±7 (150; 176)
Body weight [kg]	67±13 (49; 87)
Body mass index [kg/m^2^]	25.1±5.7 (18.2; 33.9)
ASA 1	40 (56%)
ASA 2	29 (41%)
ASA 3	2 (3%)
NYHA 1	57 (80%)
NYHA 2	14 (20%)
Serum Creatinine [mg/dL]	0.7±0.1 (0.5; 1.0)
Gamma Glutamyl Transferase [U/L]	19±14 (5; 59)
Gynaecological procedure <150 min	43 (61%)
Gynaecological procedure <300 min	23 (32%)
Major gynaecological procedure ≥300 min	5 (7%)
PONV Prophylaxis	50 (70%)
Duration of Anaesthesia [min]	154±69 (69; 314)
Interval between 2 BCM-measurements [min]*	189±68 (108; 354)
Preoperative IV Fluid Volume [mL]	301±275 (0; 1000)
Total Perioperative IV Fluid Volume [mL]	1929±866 (830; 4160)
Urinary Excretion [mL]	337±209 (100; 1000)
Net perioperative fluid balance [mL]	1600±741 (620; 3316)

Continuous variables are reported as mean ± standard deviation, (5^th^ and 95^th^ percentile); categorical variables are presented as counts and frequencies. Abbreviations: ASA = American Society of Anaesthesiologists’ physical status classification system, NYHA = New York Heart Association functional classification, PONV = postoperative nausea and vomiting, IV = intravenous. Net perioperative fluid balance = total perioperative intravenous fluid volume, corrected for urinary excretion and blood loss. *In 5 cases, the interval between the BCM-measurements was >60 minutes longer than the duration of anaesthesia, due to logistical reasons.

The average preoperative iv fluid volume administered amounted to 0.3 L, with 16 patients having received no fluid at all. The average interval between two BCM-measurements was 189 minutes and the average duration of anaesthesia was 154 minutes. During this period, patients received on average 1.9 L iv crystalloid fluid, 6 patients ≥3 L and 5 patients ≤1 L. During the interval between the two BCM-measurements, the average urinary excretion was 0.3 L, with 3 patients excreting ≥1 L. The net perioperative fluid balance amounted to 1.6 L (data shown in Table 1). PONV prophylaxis was administered in 70% of the patients.

### Vital signs, volume status and laboratory tests before and after anaesthesia

Heart rate and blood pressure were lower after anaesthesia than before, while body temperature was the same (Table 2). Before anaesthesia, no patient had fluid overload >15% ECV, and 17 patient were dehydrated <−10% ECV. After anaesthesia, one patient had fluid overload >15% ECV, and 4 patients were dehydrated <−10% ECV. Concomitantly, fluid overload increased significantly by 0.8±0.8 L, from −0.7±1.1 L preoperatively to 0.1±1.0 L postoperatively, equivalent to a 5.9±5.6% rise in ECV. This increase was paralleled by a 1.0±1.4 L rise in TBV. ICV increased only slightly without reaching statistical significance (p = 0.15).

**Table 2:** Vital signs, volume status and body composition before and after anaesthesia.

	Pre	Post	Delta	p-value
Body Temperature [°C]	*36.3±0.5*	*36.3±0.4*	*0.0±0.5*	0.71
Heart Rate [beats/min]	**82±17**	**77±17**	−**5±15**	**0.006**
Systolic Blood Pressure [mm Hg]	**135±23**	**124±22**	−**11±25**	**<0.001**
Diastolic Blood Pressure [mm Hg]	**82±12**	**70±12**	−**12±15**	**<0.001**
Mean Blood Pressure [mm Hg]	**105±17**	**92±15**	−**13±19**	**<0.001**
Extracellular Volume [L]	**13.6±1.8**	**14.4±1.9**	**0.8±0.7**	**<0.001**
Relative ECV [% body weight]	**20.8±2.3**	**22.0±2.3**	**1.2±1.0**	**<0.001**
Intracellular Volume [L]	17.3±2.4	17.5±2.2	0.2±1.1	0.15
Relative ICV [% body weight]	26.5±4.2	26.8±4.1	0.3±1.7	0.14
Total Body Volume [L]	**30.9±3.9**	**31.9±3.9**	**1.0±1.4**	**<0.001**
Relative TBV [% body weight]	**47.3±6.2**	**48.9±6.1**	**1.6±2.2**	**<0.001**
Fluid Overload [L]	−**0.7±1.1**	**0.1±1.0**	**0.8±0.8**	**<0.001**
Fluid Overload [% ECV]	−**5.1±7.5**	**0.8±6.7**	**5.9±5.6**	**<0.001**
ECV/ICV	**0.79±0.08**	**0.83±0.08**	**0.04±0.06**	**<0.001**
Serum Protein [g/L]	***71.4±6.0***	***56.5±8.1***	−***14.2±10.2***	***<0.001***
Serum Albumin [g/L]	***43.2±5.5***	***32.4±5.4***	−***12.4±5.9***	***<0.001***
C-reactive protein [mg/dL]	***0.6±1.7***	***6.2±5.8***	***5.9±5.8***	***<0.001***
Capillary Leak Index [unitless]	***2.8±9.9***	***22.1±18.9***	***25.1±20.3***	***<0.001***

Delta was calculated by subtracting any patient’s preoperative value from the postoperative value. Significance was determined by Student’s t-test (p<0.05). Italics: Data were incomplete for body temperature (number of missing values: N = 7 [pre], N = 20 [post], N = 20 [delta]), serum protein (number of missing values: N = 15 [pre], N = 39 [post], N = 46 [delta]), serum albumin (number of missing values: N = 42 [pre], N = 40 [post], N = 54 [delta]), C-reactive protein (number of missing values: N = 11 [pre], N = 39 [post], N = 42 [delta]) and Capillary Leak Index (number of missing values: N = 43 [pre], N = 43 [post], N = 55 [delta]), despite significant findings. Note that postoperative laboratory measurements were usually performed on the day after surgery (not simultaneous to the postoperative BCM-measurement). Bold: findings with p<0.05. Abbreviations: ECV = Extracellular Volume, ICV = Intracellular Volume, TBV = Total Body Fluid Volume.

Relative to the total body weight, the preoperative TBV was 47.3%, ECV 20.8% and ICV 26.5% (Table 2). The preoperative ECV:ICV ratio was 0.79. Relative TBV, relative ECV and the ECV:ICV ratio increased significantly from pre- to postoperatively, while relative ICV did not increase significantly. Serum protein and serum albumin decreased and CRP increased, from pre- to postoperatively, as manifested by a significant rise in the capillary leak index (Table 2).

### Associations between pre- to postoperative changes in volume status and perioperative fluid volume administration

**Figure 1:**
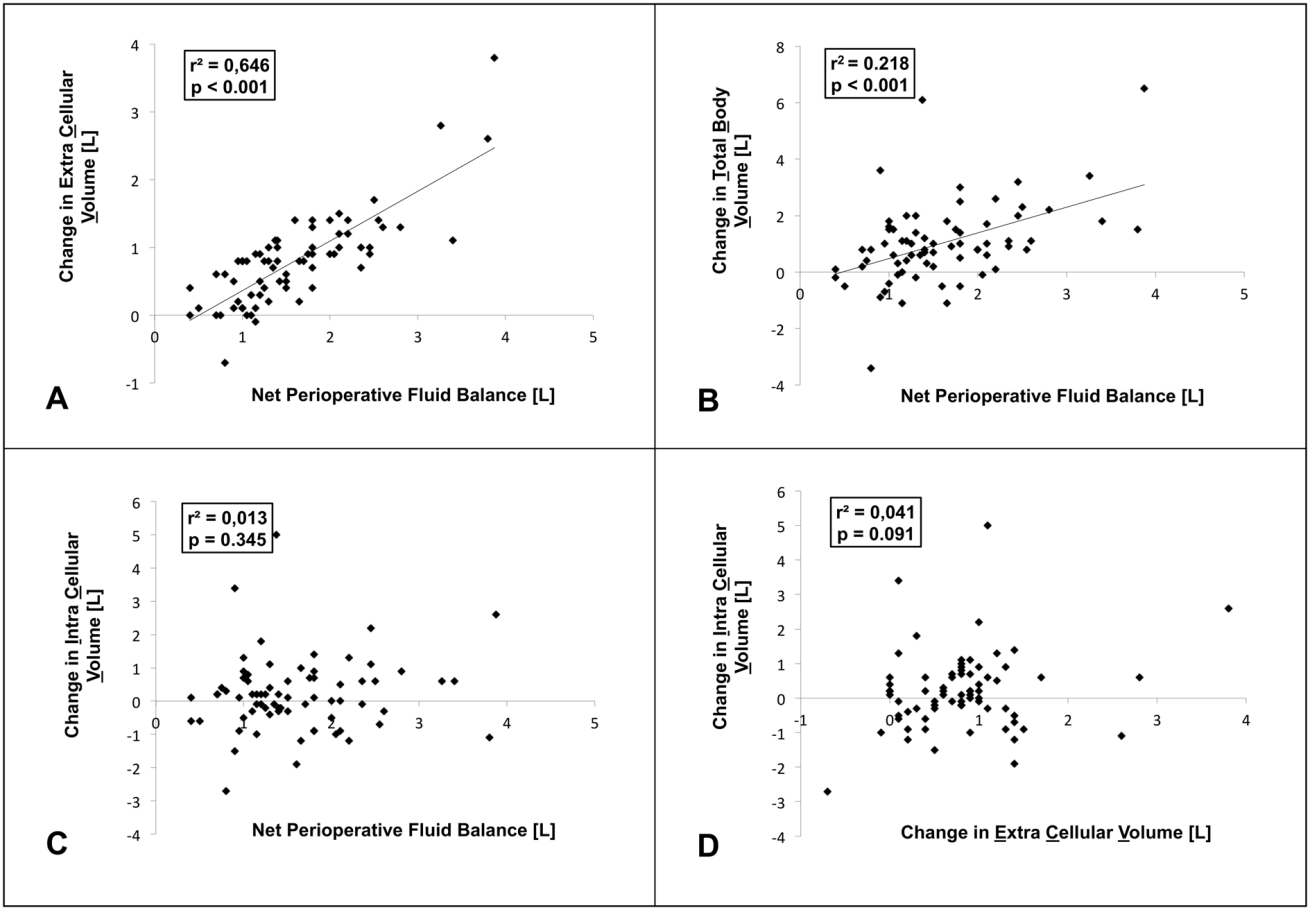
Associations between pre- to postoperative changes in volume status and net perioperative fluid balance. Scatter plots. Regression equations are as follows: **A** Change in Extracellular Volume = 0.73×Net Perioperative Fluid Balance –0.37. **B** Change in Total Body volume = 0.91×Net Perioperative Fluid Balance –0.43. **C** No linear correlation between Change in Intracellular Volume and Net Perioperative Fluid Balance. **D** No linear correlation between Change in Intracellular Volume and Change in Extracellular Volume. Pearson correlation test. R^2^ = Coefficient of determination.

We evaluated the relationship between perioperative fluid administration and changes in pre- to postoperative fluid status and identified a strong, significant correlation between the net perioperative fluid balance and change in ECV (r^2^ = 0.65, p<0.001, Figure 1A). We also identified a significant correlation between the net perioperative fluid balance and change in TBV, however, the coefficient of determination was smaller (r^2^ = 0.22, p<0.001, Figure 1B). Net perioperative fluid balance, respectively change in ECV were not significantly associated with change in ICV (Figure 1C and 1D).

### Association between net perioperative fluid balance and changes in pre- to postoperative ECV with multiple levels of adjustments

Patient-dependent parameters such as age, body mass index and renal function, might alter the relationship between perioperative fluid administration and changes in ECV. We therefore performed a linear regression analysis with net perioperative fluid balance as the predictor and change in ECV as the outcome, consecutively adjusting additionally for serum albumin, age, body mass index, serum creatinine, preoperative blood pressure, preoperative fluid therapy, pre- and postoperative capillary leak index. After stepwise adjustments, the combined effects of the predictors on change in ECV were significant in each model (Models 1–9: p<0.001). The overall model (Model 9) explained 71.3% of variance in change in ECV (r^2^ = 0.713, p<0.001, Table 3). Only net perioperative fluid balance (Beta = 0.804, p<0.001), preoperative fluid therapy (Beta = −0.152, p = 0.046) and postoperative capillary leak index (Beta = 0.202, p = 0.011) were significant individual predictors of change in ECV. The addition of all other variables altered the association only slightly. Duration of anaesthesia was not added to the model, because this variable correlated strongly with net perioperative fluid balance.

**Table 3:** Association between net perioperative fluid balance and changes in pre- to postoperative extracellular volume, with multiple levels of adjustments.

	Model 1: net perioperativefluid balance	Model 2: + serumalbumin	Model 3: + age	Model 4: + bodymass index	Model 5: + serumcreatinine	Model 6: +preoperativefluid therapy	Model 7: +preoperativeblood pressure	Model 8: +Preoperativecapillary leak index	Model 9: +postoperativecapillary leak index
Outcome variable: **Delta ECV**									
**r^2^**	0.646	0.646	0.649	0.652	0.657	0.678	0.679	0.681	0.713
**p-value^1^** for each model	**<0.001**	**<0.001**	**<0.001**	**<0.001**	**<0.001**	**<0.001**	**<0.001**	**<0.001**	**<0.001**
**p-value^2^** for individualpredictors	**<0.001**	0.845	0.479	0.435	0.330	**0.046**	0.639	0.540	**0.011**
Beta	**0.804**	0.014	−0.056	0.060	−0.077	−**0.152**	−0.049	−0.065	**0.202**

Multiple regression analysis: After stepwise adjustment the coefficients of determination (r^2^), the p-value^1^ for the combined effect of all predictors for each model, the p-value^2^ for the individual effect of a predictor and the correlation coefficient (Beta) represent the association between the outcome and the predictor variables. Boldface indicating statistical significance (p<0.05).

## Discussion

The principal findings of the present study on BCM-derived measurements performed before and after general anaesthesia in gynaecological patients were: [1] 17 of 71 patients (24%) were dehydrated (<−10% ECV), while no patient was volume overloaded (>15% ECV) before anaesthesia; [2] a net perioperative fluid balance of 1.6 L on average, administered perioperatively, led to an average increase of 0.8 L in fluid overload, respectively 0.8 L in ECV from pre- to postoperatively, but not to a significant increase in ICV; [3] concomitantly, we identified a strong, significant correlation between net perioperative fluid balance and change in ECV, which; [4] remained significant after adjustment for serum albumin, age, BMI, serum creatinine, preoperative iv fluid therapy, preoperative blood pressure and capillary leak index. Together, these data indicate that BCM-derived measurements yield plausible results on the perioperative fluid status.

Several methods of body composition analysis based on impedance are available [24], [25]. One such method is bioimpedance analysis (BIA) using single-frequency technique (SF-BIA) and another method is BIA at low and high frequency (multi-frequency MF-BIA). TBV and ECV are predicted on the basis of the primary BIA results (reactance and resistance), and ICV is calculated as the difference between TBV and ECV. SF-BIA has previously been used to detect postoperative fluid retention after major thoracic surgery [26], while MF-BIA using 4 frequencies has been employed to monitor fluid shifts after major abdominal surgery [27].

In our study we employed another impedance method, bioimpedance spectroscopy (BIS) using BCM. The BCM device measures at 50 frequencies and employs a unique body composition model for determination of fluid overload, respectively for direct prediction of TBV, ECV and ICV. Recently a study by Raimann at al. compared the precision and accuracy of SF-BIA and BIS to direct estimation methods (DEMs, i.e. deuteriumoxide-diltution, bromide-dilution and total potassium count) for body composition analysis in hemodialysis patients. In comparison to DEMs, SF-BIA and BIS were equally precise in determining TBV and ICV. However BIS-determined ECV was closer to DEM-determined ECV, in comparison to SF-BIA. According to Raimann et al., there is no real “gold standard” method with absolute accuracy for determination of the fluid status due the wide limits of agreement [28].

Another impedance-based method to estimate fluid distribution is bioelectrical impedance vector analysis (BIVA), where a vector of impedance at one frequency is evaluated against an ordinal scale with tolerance interval percentiles from a reference population [24], [25]. Body composition is analysed by the patterns of vector distribution independent of body weight. Vector displacement indicates a change in fluid status [29]. BIVA allows a ranking of repeated measurements in the individual subject compared to the reference population, but no direct prediction of TBV, ECV and ICV, which may limit its use in the clinical setting.

In the present study the values obtained for relative TBV (47.3%), relative ECV (20.8%) and relative ICV (26.5%) are in contrast to the classical textbook knowledge, stating that TBV comprise approximately 50–60% of the body weight in healthy adults [11]–[13]. Chamney et al. described strikingly similar results in 57 healthy females with a mean BMI of 24.5 kg/m^2^ measured with DEMs. Specifically, TBV determined by deuterium dilution was equivalent to 46.51% body weight, and ECV determined by bromide dilution was equivalent to 20.71% body weight [14]. Our results therefore seem to perfectly confirm the established validation of BCM-derived volume measurements against DEMs.

Mechanistically, because fluid content of lean tissue is around 73%, while fluid content of adipose tissue is around 10% [30], the proportion of lean to adipose tissue determines the proportion of TBV, ECV and ICV in relation to the body weight [31]–[33]. As the relative fat content of the whole body increases, the relative TBV decreases and the ECV/ICV ratio increases because the ECV component of adipose tissue becomes more dominant [14]. The difference in fluid content between our gynaecological study population and the average reference female described in the classical textbook seems to reflect differences in body composition and indicates that previous textbook results were generated from subjects, who were generally leaner than the present cohort, as well as those described by Chamney et al. [14].

While the bioimpedance spectroscopy-based BCM is currently used in various nephrological settings, and specifically for the management of fluid withdrawal in patients with end-stage renal disease, it has not previously been studied perioperatively. Roughly a fourth of our patients were dehydrated (ECV <−10%) and 41% of the patients had a calculated fluid deficit >1 L before surgery, although they were allowed to drink clear fluid up to two hours prior to the operation. Using BCM-derived information as a target for initial fluid management could therefore become useful to guide fluid therapy before anaesthesia in order to avoid hypovolaemia and thereby hypotension after induction [34]. On the other hand BCM could potentially assist in preventing postoperative fluid overload after major surgery.

The systemic stress response to anaesthesia and surgery is characterized by secretion of corticotrophin, antidiuretic hormone and cortisol, activation of the renin- angiotensin-aldosterone-system and stimulation of the sympathoadrenal axis to maintain hemodynamic stability and to increase salt and fluid retention [35]. Additionally, a local inflammatory reaction controlling the secretion of cytokines like interleukin 1, 6 and tumour necrosis factor augments the fluid shifts and increases vascular permeability [36]. In our cohort, we detected a marked increase in the capillary leak index due to hypoalbuminaemia and high CRP levels, most probably caused by a systemic inflammatory reaction after surgery [37], [38]. Cordemans et al. reported that capillary leak index has a biphasic course with a maximum at day 3 after shock in ventilated critically ill patients [23]. Interestingly, in the present study, the postoperative capillary leak index was a significant individual predictor of change in extracellular volume, besides net perioperative fluid balance and preoperative fluid therapy in multiple regression analysis.

Appropriate perioperative fluid therapy might limit the incidence and severity of systemic inflammation following surgery [9] and might attenuate fluid accumulation with generalized oedemas. However, for an acutely ill patient it is not uncommon to receive aggressive fluid therapy, although positive fluid balance, with postoperative fluid-based weight gain more than 10% of body weight is associated with major complications [2], [3], [5], [39]–[41]. BCM-detected fluid overload in patients with end-stage renal disease has likewise been shown to be associated with greatly increased mortality, the usual cut-off value for “hyper”-hydration and mortality being >15% ECV [16], [19], [21], [22], [42]. However, only one patient (1.4%) exceeded this cut-off value for fluid overload after anaesthesia, in this study of relatively healthy, and relatively young patients. High risk surgical patients, and especially those with poor renal function may benefit most from restrictive, intraoperative fluid management, once they have been found to be in a state of fluid overload, measured with BCM.

Our data showed a significant increase of postoperative FO, TBV and ECV, while ICV increased only slightly. It appears surprising to detect this fluid shift at all, because the gynaecological patients had normal renal function, which may leave one wondering why some of the infused volume accumulates in the periphery. Because this fluid status cannot persist indefinitely, the timing of BCM-measurements is important. In future studies, it would be interesting to measure at additional time points postoperatively to observe the return of fluid overload to normal, at least in those patients who are volume overloaded postoperatively.

A net perioperative fluid balance of 1.6±0.7 L led to a measured increase of only 1.0±1.4 L in TBV and to an increase of 0.8±0.7 L in ECV. Thus, roughly 40% of the administered fluid was “missing”. One reason for not detecting all of the administered volume in the fluid compartments of the patient may be that respiratory-, cutaneous- and wound fluid losses were not considered. Although the correlation between net perioperative fluid balance and change in extracellular fluid volume was found to be quite strong (r slightly above 0.8, since r^2^ = 0.65), predicting individual changes in fluid status after iv infusions may remain challenging in patients undergoing surgery, unless it can be clarified that an increase of only 60% is physiologically reasonable. Further methodological issues should be ruled out first.

When applying BIS technology, the bioelectrical properties are measured between the wrist and the ankle under the assumption of a steady fluid distribution. However, if the body can be thought to consist of three segments with different cross-sectional areas and lengths (arm, trunk and leg), the limbs contribute 90%, while the trunk with a large cross sectional area adds only 10% to whole body impedance [24]. After surgery, fluid has been shown to accumulate mostly in the trunk [43]. Because the same amount of fluid leads to different changes in whole-body bioimpedance, depending on whether this fluid is added to or removed from the leg, the arm, or the trunk, whole-body bioimpedance seems to be more sensitive to volume changes in the limbs than in the trunk. Segmental multi-frequency bioelectrical impedance analysis therefore might be a better approach for determination of ECV changes, because this technique is based on the assumption of a non-uniform fluid distribution within the body [44].

Pneumoperitoneum during laparoscopy increases the intra-abdominal pressure, which affects the cardiovascular system, the respiratory system and renal function [45]. Gülec et al. reported that laparoscopy can constrict the inferior vena cava leading to stasis in the peripheral veins and pooling of blood in the lower limbs [46]. In our cohort, 61% of the patients had laparoscopic surgery, which might influence the efficacy of whole body bioimpedance spectroscopy due the reduced urinary secretion, increased abdominal pressure and venous congestion in the lower limbs.

Additional study limitations: A net perioperative fluid therapy of 1.6 L would increase the body weight, but the postoperative BCM-measurement was performed with the same patient weight, because it was practically impossible to weigh the supine patient in the recovery room after surgery. Entering a patient weight of plus or minus 1.1 kg into the BCM device before performing the measurement has been shown to result in a minimal change of ECV, ICV and FO (0.08 L delta ECV, 0.01 L delta ICV and 0.04 L delta FO, respectively) [47]. Therefore, the perioperative weight difference is expected to interfere only minimally with our results, while a false weight has a higher impact on the BCM-calculated fat mass (1.1 kg higher resulting in 1.04 kg delta Fat) [47].

The present study was an open, non-interventional, observational cohort study, and as such, we were unable to compare our routinely generated BCM results with an additional reference method. Nevertheless, the BCM has been validated against direct estimation methods in more than 500 healthy subjects [14], [16], [17] and is now widely accepted as a very precise whole body bioimpedance spectroscopy device [48]–[51]. Future studies, for example using segmental bioimpedance may help clarify whether another bioimpedance method is even better suited to assess the volume status in patients undergoing surgery.

## Conclusion

In conclusion, the results of the present study, obtained with whole body bioimpedance spectroscopy using BCM, show that intraoperative crystalloid fluid administration leads to a significant, and clinically meaningful increase of the extracellular volume compartment, while the intracellular fluid volume increased only slightly. The BCM might serve as a simple and non-invasive device for objective assessment of body fluid distribution in the perioperative setting. Further research is now required to evaluate prospectively, whether BCM-guided fluid administration may improve the postoperative volume status, as well as clinical outcomes in surgical patients.
