# Correction: Bioimpedance Spectroscopy for Assessment of Volume Status in Patients before and after General Anaesthesia

**DOI:** 10.1371/journal.pone.0117956

**Published:** 2015-03-09

**Authors:** The PLOS ONE Staff

There are errors in the “Results” section in the Abstract of the published article. Duration of anesthesia should read 154 ± 69 minutes, and the value for perioperative urinary excretion should read 0.3 ± 0.2 L.

The headings for Table 3 are misaligned. Please view the correct Table 3 here.

**Table 3:** Association between net perioperative fluid balance and changes in pre- to postoperative extracellular volume, with multiple levels of adjustments.

	**Model 1**: net perioperative fluid balance	**Model 2**: + serum albumin	**Model 3**: + age	**Model 4**: + body mass index	**Model 5**: + serum creatinine	**Model 6**: + preoperative fluid therapy	**Model 7**: + preoperative blood pressure	**Model 8**: + preoperative capillary leak index	**Model 9**: + postoperative capillary leak index
Outcome variable: **Delta ECV**									
r^2^	0.646	0.646	0.649	0.652	0.657	0.678	0.679	0.681	0.713
**p-value^1^** for each model	**<0.001**	**<0.001**	**<0.001**	**<0.001**	**<0.001**	**<0.001**	**<0.001**	**<0.001**	**<0.001**
**p-value^2^** for individual predictors	**<0.001**	0.845	0.479	0.435	0.330	**0.046**	0.639	0.540	**0.011**
Beta	**0.804**	0.014	−0.056	0.060	−0.077	**−0.152**	−0.049	−0.065	**0.202**

Multiple regression analysis: After stepwise adjustment the coefficients of determination (r^2^), the p-value^1^ for the combined effect of all predictors for each model, the p-value^2^ for the individual effect of a predictor and the correlation coefficient (Beta) represent the association between the outcome and the predictor variables. Boldface indicating statistical significance (p<0.05).

## References

[1] Ernstbrunner M, Kostner L, Kimberger O, Wabel P, Säemann M, Markstaller K, et al (2014) Bioimpedance Spectroscopy for Assessment of Volume Status in Patients before and after General Anaesthesia. PLoS ONE 9(10): e111139 doi:10.1371/journal.pone.0111139 25360698 10.1371/journal.pone.0111139 PMC4215896

